# CO-CREATING A VIRTUAL REALITY IN HOSPITAL WITH PATIENT AND FAMILY PARTNERS AND STAFF FOR OLDER ADULTS WITH DEMENTIA

**DOI:** 10.1093/geroni/igae098.0957

**Published:** 2024-12-31

**Authors:** Lillian Hung, Ben Mortenson, Angelica Lim, Jennifer Boger, Jim Mann, Lily Wong, Haopu (Lily) Ren, Albin Soni

**Affiliations:** University of British Columbia, Vancouver, British Columbia, Canada; University of British Columbia, Vancouver, British Columbia, Canada; Simon Fraser University, Burnaby, British Columbia, Canada; University of Waterloo, Waterloo, Ontario, Canada; University of British Columbia, Vancouver, British Columbia, Canada; University of British Columbia, Vancouver, British Columbia, Canada; University of British Columbia, Vancouver, British Columbia, Canada; University of British Columbia, Vancouver, British Columbia, Canada

## Abstract

Growing evidence suggests that Virtual Reality (VR) is promising to improve the wellbeing of older patients in dementia care units in hospitals. However, older patients are often excluded from VR opportunities. Engaging patient partners, family caregivers and staff in co-creating VR program shows potential in addressing unique needs of older patients, supporting staff in implementation, and enhancing understanding complexity in clinical settings. However, literature that describes fulsome partnership with abovementioned joint stakeholders in the co-creation process is absent. The study aims to understand psychosocial needs of older adults with dementia in hospital and how VR could be best implemented in the complex clinical setting. Drawing principles of Collaborative Action Research (CAR) and applying an equity and inclusive lens, we conducted qualitative focus groups, co-design workshops and interviews with 46 stakeholders (7 patient partners, 8 family caregivers, 19 staff members and 12 leaders) in hospital. Consolidated Framework for Implementation Research (CFIR) informed our data collection and analysis. We identified three key themes to co-create a VR program for older adults with dementia in hospital to address their psychosocial needs and facilitate staff’s implementation, acronymized as

**aim:**

1) Approach matters; 2) Interactiveness; and 3) Multi-sensory stimulation. Our results underscore the imperative of engaging joint stakeholders in co-creation for older adults with dementia in hospital to address their psychosocial needs with VR, who deserve digital equity but are traditionally underrepresented in technology program development and implementation. This study contributes valuable insights into the future development and deployment of VR in geriatric care settings.

